# Non-Hodgkin’s Lymphoma Presenting as a Huge Ocular Adnexal and Forehead Mass

**Published:** 2011-01

**Authors:** Afekhide Ernest Omoti, Caroline Edijana Omoti, Osesogie Usualele Ogbeide

**Affiliations:** University of Benin Teaching Hospital, Benin City, Nigeria

**Keywords:** Non-Hodgkin’s Lymphoma, Forehead Mass, Lid Mass

## Abstract

**Purpose:**

To report a case of non-Hodgkin’s lymphoma (NHL) presenting as an ocular adnexal and forehead mass.

**Case Report:**

An elderly male patient was referred by a neurosurgeon to the eye clinic with a six-month history of a massive tumor measuring 12×16×8 cm involving the right side of the forehead, eyebrow and upper eyelid. Neurological examination had been normal and computed tomography revealed no intracranial extension. The patient was referred to an otorhinolaryngologist who performed an incisional biopsy which revealed the mass to be NHL. He received chemotherapy with CHOP regimen (cyclophosphamide, adriamycin, vincristine and prednisolone) resulting in reduction in lesion size leaving a phthysical eyeball and a ptotic lid.

**Conclusion:**

Non-Hodgkin’s lymphoma may occur in almost any part of the body and should be considered in the differential diagnosis of extralymphoid tumors.

## INTRODUCTION

Non-Hodgkin’s lymphoma (NHL) comprises 90% of all hymphomas.^1^ The usual sites of involvement are lymph nodes but all ocular tissues and adnexa may become affected.^2^ Lymphomas of the eye and its adnexa are frequently of B cell lineage but may rarely be non-B-cell NHL.^3^ The lack of pathognomonic features, high clinical variability, and the limited value of imaging techniques and histopathological measures often lead to delays in diagnosis.^4^

Lymphoma may present as a localized eyelid mass or nodule.^3^,^5^ These mass lesions are usually small and may cause ptosis. The patient reported herein presented a diagnostic dilemma. Initial presentation was a massive tumor involving the forehead, eyebrow and upper eyelid and the diagnosis was reached only after incisional biopsy.

## CASE REPORT

A 63-year-old male subject, a retired trader, presented to the University of Benin Teaching Hospital, Benin City, Nigeria, with a mass lesion involving the right side of the face of 6 months’ duration. There was no history of trauma, but the patient had experienced weight loss.

**Figure 1:**
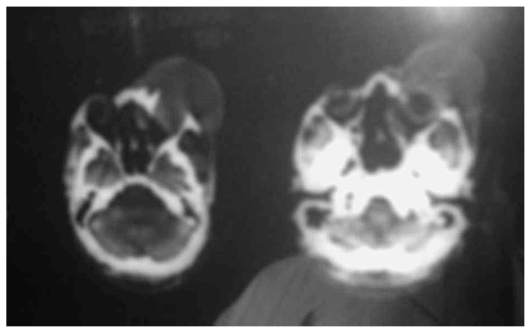
Cranial CT scan of the patient with right sided forehead and ocular adnexal mass showing normal ocular dimensions without any intracranial connection.

He had first presented to a general hospital from where he was referred to a neurosurgery center to rule out intracranial lesions. He underwent cranial computed tomography (CT) scanning which showed that the mass had no intracranial connection or extension and that the eyeball was intact (Fig. 1). He was then referred to our clinic.

**Figure 2:**
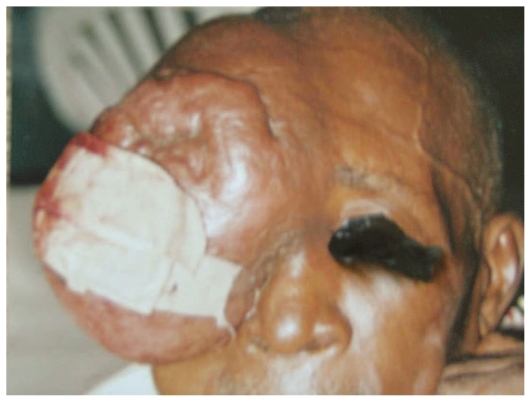
The patient with massive right sided forehead and ocular adnexal mass after incisional biopsy.

On ophthalmologic examination, the patient was not in distress, but the mass was very large involving the right side of the forehead, right eyebrow and the right upper eyelid such that it covered the cheek and extended down to the nostrils. The right eyeball could not be visualized (Fig. 2). The mass measured 12×16×8 cm. Visual acuity was light perception in the right eye and 20/30 in the left eye. Examination of the left eye was unremarkable; the pupil was round, central and reactive to light. There was no palpable preauricular, postauricular, submandibular, cervical or peripheral lymphadenopathy at the time of presentation. Neurological and other systemic examinations were normal. Considering CT scan findings, it was assumed that the problem may be arising from the frontal sinus, therefore the patient was referred to an otorhinolaryngologist who performed an incisional biopsy on the mass.

**Figure 3:**
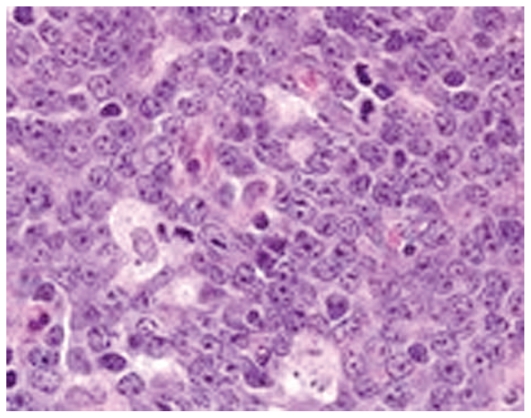
Histologic section shows small lymphocytes with mildly pleomorphic vesicular nuclei, arranged diffusely.

Histology of the biopsy specimen disclosed sections of neoplastic tissue made up of small lymphocytes with mildly pleomorphic vesicular nuclei, arranged diffusely; most had plasmacytoid nuclei (Fig. 3). A diagnosis of non-Hodgkin’s lymphoma was made.

**Figure 4:**
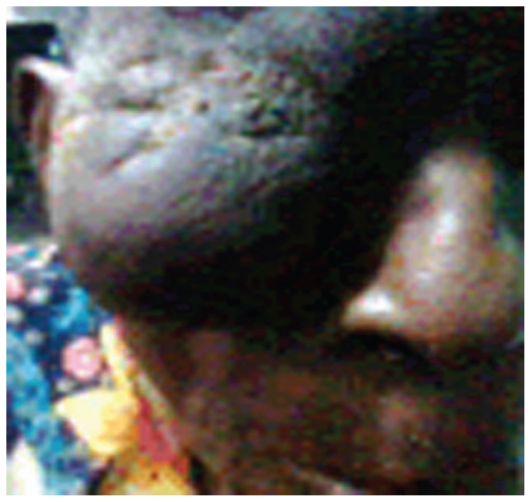
The same patient after the second course of chemotherapy; considerable reduction occurred in the size of the adnexal mass.

The patient was then referred to the hematology department, where chemotherapy was commenced after a complete workup. He was placed on CHOP regimen (cyclophosphamide 650 mg/m^2^ IV, days 1 and 8; adriamycin 45 mg/m^2^ IV, days 1 and 8; vincristine 1.5 mg/m^2^ IV, days 1 and 8 and prednisolone 20 mg PO, TDS). The mass was considerably reduced in size after the second course of chemotherapy (Fig. 4); after six courses it resolved completely, leaving a ptotic upper eyelid and a phthisical eyeball.

## DISCUSSION

Non-Hodgkin’s lymphoma has a wide variety of ophthalmic and neuro-ophthalmic presentations.^2^ This particular case was seen by a neurosurgeon, an ophthalmologist and an otorhinolaryngologist, but none of them suspected lymphoma. It was only after the incisional biopsy that the true nature of the mass was revealed and he was referred to a haematologist for treatment.

A review of the literature revealed no case as dramatic as ours; only 3 reports of lymphoma were somehow similar to this presentation.^6^–^8^ The closest case was a 74-year-old woman complaining of a growing mass in her forehead where she had sustained minor trauma one month previously. The mass measured 6×6×2 cm and was found in the right frontal region. CT scan showed a large soft tissue mass in the subcutaneous tissue and a small mass in the ethmoid sinus, with erosion in the inner and outer tables of the frontal bone. Histopathological examination showed the tumor to be a malignant lymphoma of non-Hodgkin and diffuse mixed cell type. Systemic investigations including 99mTechnetium-MDP bone scan, 67 Gallium citrate scan, bone marrow aspiration, and CT scanning, disclosed no evidence of systemic lymphoma. The patient received chemotherapy with cyclophosphamide, doxorubicin HCl, vincristine sulfate and prednisolone, leading to complete remission. 7

The second report described a 72-year-old man with a steadily growing mass (4×4×2 cm) in the frontal region which was noticed following head injury. Six months earlier, he had declined treatment for malignant lymphoma (small non-cleaved cell type), discovered upon evaluation of a subcutaneous mass in his forearm. Computed tomography disclosed a large, homogeneously enhancing mass with both extradural and extracranial extension which had destroyed the left frontal bone. The skull tumor was completely resected and a diagnosis of malignant lymphoma, large-cell, immunoblastic and plasmacytoid type, was confirmed.^7^

The third case was a 65-year-old male subject who complained of a huge and rapidly growing scalp mass (largest dimensions 13×9×4 cm) and hemiparesis. The craniogram revealed a large osteolytic lesion in the right parietal bone. The histological diagnosis was malignant lymphoma (B cell type).^8^

Although there are a few other reports of lymphoma affecting the skull, only the first two of the abovementioned cases, presented in the frontal area but were not large enough to affect the upper lid and cover the cheek. The current report shows that NHL may present at unusual sites and pose a diagnostic dilemma. Such cases can cause delays in initiating definitive treatment with potentially disastrous consequences for the patient.^4^ Therefore, NHL should be considered in the differential diagnosis of extralymphoid mass lesions at any location.
